# DIVERSITY OF EXPERIENCES OF AGING AMONG BIRTH COHORTS AND RACIAL/ETHNIC GROUPS IN NSHAP

**DOI:** 10.1093/geroni/igac059.543

**Published:** 2022-12-20

**Authors:** Linda Waite, Amelia Karraker

## Abstract

Aging, the process of getting older, takes many forms. This symposium uses data from the National Social Life, Health, and Aging Project to study changes and differences between groups in the experience of older adults. Dale and coauthors look at the implications for future mortality of failing to respond to a follow-up interview in an ongoing survey. Those who drop out of NSHAP are more similar to those who died than to those who are reinterviewed, suggesting that survey dropout carries important information about health that has been ignored to this point. Choi and Waite compare social networks and social support for the Silent Generation cohort, born 1920-1947, to the Baby Boom cohort, born 1947-1965, and finds that the BB cohort has fewer kin in their networks and receive less support from family and friends than the older cohort, suggesting that they are more disconnected. Piedra and Iveniuk compare social networks of White, Black and Hispanic older adults. They find that Hispanics are initially more likely to have restricted networks, showing low social connectivity but increase in network diversity over time, suggesting resilience, whereas White and Black move toward lower connectivity as they get old. Iveniuk and Gupta compare marital histories of older adults in different racial and ethnic subgroups, with Blacks least and Whites most likely to be married at older ages, and Hispanics more likely to be recently widowed. These presentations point to the diversity of pathways through aging, and their consequences.

